# Physicochemical Characterization of Thermally Processed Goose Bone Ash for Bone Regeneration

**DOI:** 10.3390/jfb14070351

**Published:** 2023-06-30

**Authors:** Fatimah Suhaily Abdul Rahman, Abdul Manaf Abdullah, Asanah Radhi, Wan Nazatul Shima Shahidan, Johari Yap Abdullah

**Affiliations:** 1 School of Dental Sciences, Health Campus, Universiti Sains Malaysia, Kubang Kerian 16150, Kelantan, Malaysia; fsuhaily@gmail.com (F.S.A.R.); shima@usm.my (W.N.S.S.); 2 School of Mechanical Engineering, College of Engineering, Universiti Teknologi MARA, Shah Alam 40450, Selangor, Malaysia; abdulmanaf@uitm.edu.my; 3 Faculty of Bioengineering and Technology, Universiti Malaysia Kelantan, Jeli 17600, Kelantan, Malaysia

**Keywords:** goose bone, bioceramic, calcium phosphate, bone regeneration, physicochemical property, hydroxyapatite

## Abstract

Goose bone is traditionally applied for many ailments including bone fractures. Goose bone that consists of calcium phosphate plays a major role in bone regeneration. In this study, the production of goose bone ash (GBA) was translated from a traditional process into one of a laboratory scale via thermal and mechanical methods. The GBA was thermally processed via calcination at 300 °C and 900 °C. The differences in physicochemical properties between studied GBA (SGBA) and commercial GBA (CGBA) were elucidated via Fourier transform infrared (FT-IR), X-ray fluorescence (XRF), X-ray diffraction (XRD) and electron diffraction X-Ray (EDX). The morphological properties of SGBA and CGBA were characterized using field emission scanning electron microscopy (FESEM) in which nano-sized particles were detected. The results showed that the SGBA of 300 °C had comparable physicochemical properties to those of CGBA. A high processing temperature was associated with decreasing organic compounds and increasing crystallinity. The finding from EDX suggests that sintering at 900 °C (SGBA 900) demonstrated the presence of hydroxyapatite in the mineralogical phase and had a Ca/P atomic ratio of 1.64 which is comparable to the ideal stoichiometric ratio of 1.67. Findings from this study could be used for the further exploration of GBA as a potential material for bone regeneration via the elucidation of their biological properties in the next experimental setting.

## 1. Introduction

Bone is one of the natural bioceramics that contains calcium phosphate as a potential material for improving the treatment of bone disease. An injured bone can be easily healed due to the high capacity of bone regeneration, but if the injuries exceed a critical size, a scaffold or template is required to induce the growth of bone tissue [1]. For this purpose, goose bone was selected as one alternative scaffold with which to regenerate defective bone tissue. The utilization of goose bone as a traditional remedy is widely used in Malay culture as it can help in the healing of bone fractures due to its mineral content. Other than that, it was believed that a consistent consumption of goose bone in daily life could boost the immune system [2].

Avian species such as goose have a unique type of bone that is mainly found in their females, known as medullary bone. Medullary bone is a layer of material found inside a regular bone in birds. It forms a porous and spongy layer inside the bones of females when they are going to lay eggs. In terms of mineral content, the skeleton consists of a calcium-rich medullary bone. The formation of medullary bone was induced via the use of estrogenic and androgenic hormones during the egg laying cycle [3]. During the oviposition cycle, the demand for calcium is higher and calcium metabolism is largely derived from dietary sources and skeletal reserves [4]. To accommodate the increased calcium demand from the bone, the medullary bone is made without a mechanical function [5]. This spongy bone material supplies a labile calcium source for eggshell formation and is located within the medullary cavity of the mid-diaphysis of long bones, particularly those of the legs [4]. From previous reports, the source of calcium is one of the crucial ingredients that must be in the bioceramic scaffold [6,7]. Therefore, the utilization of unique avian bones such as goose bone may present a great opportunity to develop beneficial calcium phosphate-based material to fill the gap of the current issue in bone regeneration treatment.

Generally, normal bone tissue is made up of type 1 collagen and hydroxyapatite (HAp), which are highly compatible with bone functions throughout the body [8]. These structured, mineralized and elastic natural materials support and protect other tissues in the body system. The ideal bone repair material must possess good biocompatibility to maximize the osteogenesis process and good degradability to accelerate the regeneration of bone. These characteristics are the requirements for the implantation site of the scaffold [8,9,10].

Calcium phosphate-based material is biocompatible, osteoconductive, and osteoinductive, properties which are favored in medical industry, particularly in orthopedics, dentistry, and the coating application of metallic implants [11]. This bioceramic material is densely structured with textured granulated fillers suitable for bone reconstruction due to its similarity with the natural mineral composition of bone tissue. HAp, Ca_10_ (PO_4_)_6_ (OH)_2_, is the main mineral component of bones and teeth; it occurs naturally in calcium phosphate-based material. The physical and chemical properties of natural hydroxyapatite and bone are found to be identical as both of them have a porous structure, that makes them biocompatible [12]. HAp can be obtained naturally or synthetically, and the need for this material depends on the medical applications due to its different physicochemical properties. Nowadays, the production of nanohydroxyapatite (nano-HA) has drawn significant interest in the field of bone regeneration. The utilization of nanoparticles in many applications including bone regeneration therapy is an added advantage. Surface properties behave differently at the nanoscale and due to these properties, nanoparticles can improve the surface functionalization that is useful in medical treatments.

Natural HAp can be derived from natural sources such as animal bones, scales, shells, and mineral materials, which are basically composed of calcium, as the major constituents. The production of natural HAp is non-stoichiometric since trace elements such as Na, Zn, Mg, K, Si, Ba, Fe, and F, and ions such as CO_3_^2−^, were found in the organic materials [13]. Basically, these impurities affect the content of calcium and hence produce a higher HAp ratio (>1.67) compared to that of stoichiometric HAp. The chemical composition of natural HAp resembles that of the inorganic part of structured bone, which is beneficial for rapid bone regeneration due to its richness of anions and cations [14]. The presence of ion substitution in the apatite structure affects the crystallinity phase, which influences the dissolution rate, significantly enhances the cell proliferation of human osteoblast cells and eventually promotes osseointegration [15].

It has also been reported that the existence of amino acid sequences in the collagen of structured bone helps cell growth by binding the cells and initiating signal transduction [16,17]. Other organic sources in the bone skeleton, including fibronectin and laminin, have a biological property that regulates cell growth upon contact with cells [16]. Collagen type I in the bone matrix is the most abundant extracellular matrix (ECM) protein and is a crucial material for bone strength. A previous study demonstrated that the interaction between bone marrow cells and collagen type I led to their differentiation into osteoblast cells via an increase in osteogenic gene expression and an elevation alkaline phosphatase activity [18].

Previously, several studies have shown the usage of different animal bone ashes for bone regeneration therapies [19,20,21]. The efficacy of bone ashes as scaffolds depend on the bone composition ratios. These ratios determine the mineral content of thermally processed bone ash. The processing temperature plays an important role, resulting in the mineralogical structure of the calcified bone. As reported, the crystallinity of the bone ash increases as the temperature increases. In mammals such as porcine animals, cancellous bone particles demonstrated a larger surface area and greater pore volume at a lower burning temperature (below 400 °C) than at a high temperature (above 1200 °C) [20]. A denser structure achieved at a high temperature may offer a good property for bone scaffold, biologically. In other species such as ovine species, cortical bone revealed an increase in crystallinity with increased temperature and a longer processing time [21]. Similar characteristics were observed for goose bone ash at a high burning temperature, except the structure of goose bone is more porous than that of the other bones. This structure provides a greater surface area with a larger pore size that is potentially good for osteoblast and bone regeneration [22]. The purpose of this research is to provide in-depth observations of the differences in physicochemical and morphological properties between CGBA and SGBA via XRF, FTIR, XRD and FESEM. The SGBA was subjected to two different heating temperatures, i.e., 300 °C and 900 °C. The heating temperature of 300 °C was selected based on a common approach for the deproteinization of bone which involves a low processing temperature [23]. Meanwhile, the selection of 900 °C is based on a previous study which mostly suggested the ideal sintering temperature for HAp production from natural bone to be in the range of 900–1200 °C [24]. These results will pave a way for the investigation of goose bone ash potential as an alternative material with which to promote bone regeneration. 

## 2. Materials and Methods

### 2.1. Preparation of Goose Bone Ash (GBA)

Goose bones which were poultry waste were collected from a farmhouse located in Pasir Mas, Kelantan, Malaysia. The goose bones were collected from the waste of adult geese slaughtered for commercial purposes. In this study, two types of bone samples were used, which were commercial GBA (control group) from Roxhana Ventures Sdn. Bhd. and the studied GBA (study group). The studied GBA (SGBA) is a bone ash that is produced at the laboratory scale. The SGBA was prepared by washing the raw bones, cutting them into small pieces and finally drying them in the oven for 4 h. Eventually, the ash bone sample was obtained via the calcination of the bones at 300 °C and 900 °C using a furnace (Daihan Wise Therm, Seoul, Republic of Korea) for 3 h. There were two types of SGBA samples (300 °C and 900 °C) and the physicochemical characterizations of these materials were executed and were compared to those of CGBA.

### 2.2. Physicochemical Characterizations

#### 2.2.1. X-ray Fluorescence (XRF) Measurement

The CGBA and SGBA elemental analyses were conducted using Rigaku Supermini 200 Wavelength Disperse X-ray F Spectrometer (WDXRF). The elemental oxide composition of the ash bone samples was determined. The value of each element was quantified as a percentage and the value was calculated based on mass.

#### 2.2.2. Fourier Transform Infrared (FTIR) Measurement

The functional groups in the GBA were detected via FTIR spectroscopy. The ash samples were mixed with potassium bromide (KBr) powder and crushed in a mortar before pellets were made. The samples were analyzed with Perkin Elmer. Data spectra were collected in the absorption mode between 4000 and 400 cm^−1^ with a resolution of 1 cm^−1^.

#### 2.2.3. X-ray Diffraction (XRD) Measurement

The X-ray diffraction patterns were obtained using Rigaku Smartlab X-Ray Diffractometer (Tokyo, Japan) with Cu Kα radiation (λ = 1.5406 Å), at an accelerating voltage of 30 kV and a 20 mA current, and the measurement was performed at room temperature. Diffractograms were recorded from 10–80° on a 2θ scale with a rate of 10° per minute. Prior to the analysis, the GBAs samples were oven-dried at 60 °C overnight. Then, the powder samples were ground with a pestle and pressed into a disc pellet with a 30 mm diameter. The samples were inserted in the vacuum and analyzed for 20 min. The XRD analysis determined the changes in the mineral phase of bone ash during heating or burning. The amorphous and crystalline phase of the samples generalized the pattern of mineral phases.

#### 2.2.4. Field Emission Scanning Electron Microscopy (FESEM) Measurement with Electron Diffraction X-ray (EDX)

The morphology, shape, and texture of the GBAs were analyzed via field emission scanning electron microscopy (FESEM) (Quanta 450 FEG, Fei, Eindhoven, The Netherlands). The GBA powders were stored in a desiccator prior to the analysis. Then, the samples were mounted on the SEM specimen stubs and coated with a thin layer of gold using an ion sputtering device (Leica EM SCD 005, Prague, Czech Republic) at current and vacuum values of 20 mA and 0.05 mbar, respectively. The morphology of GBAs was observed using FESEM at magnifications of 500 and 100,000.

## 3. Results and Discussion

### 3.1. XRF Analysis

The elemental comparison between GBAs is outlined in Table 1. The differences in the elemental content were calculated using mass (%). In the X-ray fluorescence analysis, it was revealed that calcium (Ca) was the main constituent in GBAs with an average of 75% to 81%. The SGBA 300 demonstrated the highest value of 80.6% compared to those of SGBA 900 and CGBA which were 77.7% and 75.7%, respectively. In addition, other major elements that were generally found in bone such as phosphorus (P), potassium (K), chlorine (Cl), ferrous (Fe), magnesium (Mg) and sulfur (S) were detected in all GBA samples except for SGBA 300 in which no Mg was detected.

Based on the results, there were differences in the element contents of the GBA samples, such as those of Ca, P, and Mg. This could be because of the type of goose bone that was utilized as the raw material in producing bone ash. There was no information provided about the breed and the exact age of the obtained goose bones from the local slaughterhouse. These goose attributes can influence the hydroxyapatite composition of calcified tissues [25]. A similar study by Bahrololoom et al. successfully demonstrated the detection of numerous elements in cortical bone from cattle [26]. The variations of elements in bone noticeably rely on some biological factors such as nutrition [27].

In addition to having Ca and P as its main constituents, GBA contains small amounts of inorganic elements such as Mg, Na, K, Zn, and Sr in the HA structure. Apatite replacement with the aforementioned inorganic ions is known to have a significant impact to such an extent that it affects biological HAp functions. The introduction of Mg impurities into HAp structures has been demonstrated in earlier research to help promote osteoblast proliferation, which is essential for the generation of bone [28].

Ca is one of the ions that are mostly found in the bone matrix, and is mainly found in the form of calcium phosphate [7]. From Table 1, the element phosphorus (P) shows a range from 16% to 21% across all samples in which SGBA 900 exhibited the highest value (20.2%). A previous study showed that a high concentration of calcium ions from bone can induce the cell proliferation of osteoblasts and help osteoblastic activity for bone regeneration [29]. Meanwhile, phosphorus is an essential element to maintain biological structure and also bone mineralization [30].

The phase composition of GBA (Table 2) shows the content of calcium, phosphorus and magnesium (in oxide forms) of natural HAp extracted from goose bone. The range of CaO in all GBA samples lies between 63% and 67%, and this result is somewhat higher than that for the CaO found in bovine bone that was only around 53.4% [26]. The higher composition of Ca in GBA might be due to the presence of medullary bone in female avian species that is rich with Ca content as a labile source. Based on the phase composition of CaO and P_2_O_5_, the calculated values of Ca/P for CGBA, SGBA 300 and SGBA 900 were 1.99, 2.30 and 1.87, respectively.

### 3.2. FTIR Analysis

**Figure 1:**
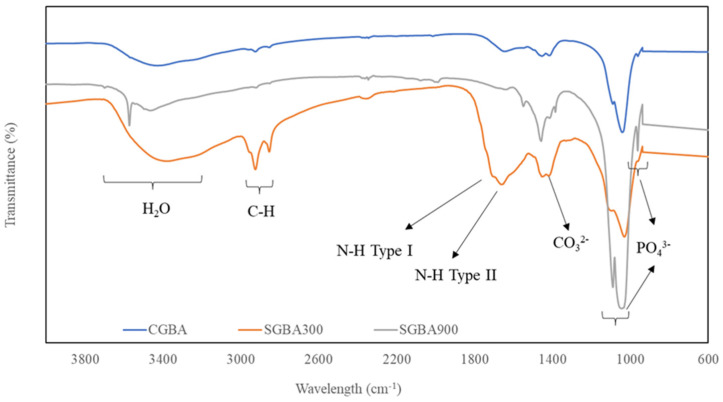
FTIR spectrum of CGBA and SGBA.

Basically, calcium phosphate-based materials such as GBA were determined according to their Ca/P molar ratio, but from a chemistry perspective, they are formed of three main elements: calcium, phosphorus, and oxygen. In Figure 1, the FTIR analysis of GBA shows the characteristic bands associated with functional groups PO_4_^3−^, OH^−^ and CO_3_^2−^. In general, the highest intensity and sharpness of the peaks were due to the phosphate and hydroxyl group which was the highest in SGBA 900 compared to that in SGBA 300 and CGBA, indicating the increased crystallinity of HA. The spectrum also shows that multiple peaks of phosphate ions were detected which were 1052 cm^−1^ (asymmetric), 604 cm^−1^ and 548 cm^−1^ (bending). The absorption bands of CGBA specifically appeared at 1037 cm^−1^ (asymmetric stretching) and at 603 cm^−1^ and 564 cm^−1^ (bending). Meanwhile, the presence of phosphate ions for SGBA 300 were detected at 1031 cm^−1^ (asymmetric) and 604 cm^−1^ and 562 cm^−1^ (bending).

The displayed bands at approximately 2854 cm^−1^ and 2924 cm^−1^ (Figure 1) were obviously detected for SGBA 300, but the bands were visible for the rest of the bone ashes. These bands could be attributed to asymmetric C-H bonds in the aliphatic chains of collagen, particularly those of the amide group [31]. A previous study reported that three types of amide absorption bands were identified from the processed natural bones: amide I (1600–1700 cm^−1^; C=O stretching vibrations), amide II (1500–1550 cm^−1^; N-H deformation and amide III (1200–1300 cm^−1^; N-H deformation and other complex modes resulting from a mixture of several coordinate displacements) [31].

The detection of the absorption band of amide type I at 1706 cm^−1^ and amide type II at 1508 cm^−1^ of SGBA 300 was observed but, not for SGBA 900, and this result was similarly found in a previous study [32]. Meanwhile, the absorption band of amide (type II) for CGBA was slightly detected. However, all GBA samples did not reveal any absorption band for amide (type III) formation. The previous study reported that when the amide group was associated with a water molecule, the formation of a hydrogen bridge occurred; therefore, the absorption band in a range of 3330 cm^−1^–3425 cm^−1^ were very noticeable for SGBA 300 [33]. This result may indicate the co-existence of an inorganic part (calcium phosphate) and organics traces in goose bone ashes [32]. As reported, bone tissue is composed of 60% inorganic components (hydroxyapatite), 30% inorganic components (bone matrix protein) and 10% water [34].

Functional analysis for SGBA 300 revealed that the thermal processing of the sample did not fully destroy the organic part and that it was considered hybrid organic–inorganic material. Additionally, the FTIR analysis of CGBA demonstrated the reduction property of the organic component compared to that in goose bone calcified at 300 °C. This occurrence of this phenomenon might be due to the unknown traditional processing method of CGBA, particularly in the thermal aspect which might be due to a secrecy issue of the industry. However, it can be speculated that CGBA was processed either at a higher temperature or with several more stages of the heating process than those used for processing SGBA 300 based on the current analysis. A small peak at 1414 cm^−1^ which corresponds to that of the carbonate groups (CO_3_^2−^) was detected for CGBA and SGBA 300 but not for SGBA 900, confirming the isolation of carbonated HA from the goose bone at 900 °C. The amount of the remaining organic material is crucial as it determines the ability to safely use grafting materials without provoking any immunological reactions [35]. In addition, the absorption bands of amide groups are also no longer visible for SGBA 900, indicating the degradation of the collagen structure [36]. Regardless of the intensity of the peaks, both SGBA samples demonstrated a similar fingerprint region of the IR spectra to that of CGBA where the absorption bands below 1500 cm^−1^ indicated the presence of molecules in the mineral part of bone.

### 3.3. XRD Analysis

**Figure 2:**
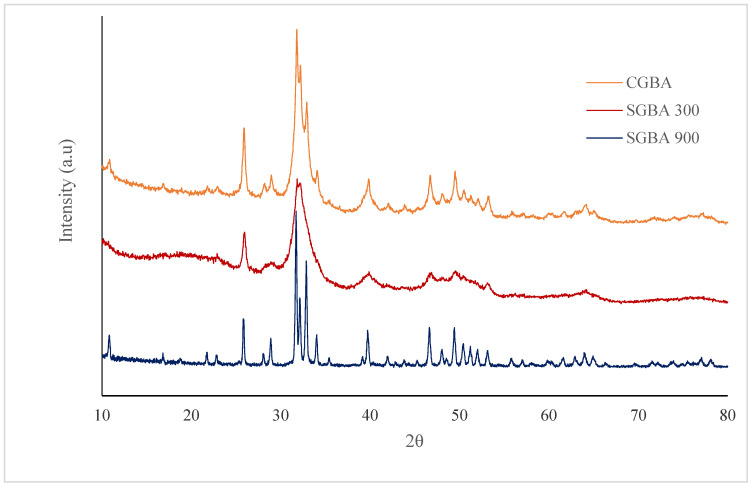
XRD spectra for commercial (CGBA) and calcinated (SGBA 300 and SGBA 900) goose bone ash.

The phase identification of GBA samples was performed using X-ray diffraction. Figure 2 demonstrated the XRD patterns of commercial goose bone ash and calcinated goose bone at temperatures of 300 °C and 900 °C. The crystalline nature and phase composition were confirmed via the XRD analysis. The obtained XRD spectra were compared to those in the COD 9,002,214 standard HAp data. As shown in Figure 2, all the crystalline peaks in the XRD spectra closely matched with the peaks in standard HAp. This means that the thermal process produced natural HAp. The XRD results also suggest that the HAp present in the goose bone matrix was not disrupted by calcination at up to 900 °C.

It is evident that when the calcination temperature was increased to 900 °C, the intensity of the diffraction peaks increased, and the peaks sharpened and narrowed. This may have been due to the crystal size and crystalline nature becoming more prevalent at a higher temperature. The CGBA and SGBA 300 that was calcined at 300 °C displayed larger peaks with a low intensity, indicating that the organic material remaining in the bone matrix had not completely been removed.

Heating at a lower temperature, such as 300 °C, produces broad diffraction peaks that correlate to poor-crystallinity apatite, possibly due to the presence of a low concentration of carbonated groups in the sample. This is indicated by the FTIR spectrum of SGBA 300 (Figure 1) that exhibited that the band of carbonate ions is at 1000 which is stronger and broader, indicating that an increase in carbonate ions causes a decrease in the crystallinity of the structure [37]. Spence et al. reported that carbonate hydroxyapatite structured material accelerates osteogenesis by enhancing bioresorption [38]. By increasing the heating temperature to 900 °C, the diffraction peaks become more intense, sharper, and narrower, suggesting an increase in crystallinity and crystal size. This suggests that as the temperature increased to 900 °C, the raw, amorphous goose bone transformed into a crystalline phase with a diminishing organic phase and carbonates [39]. This is supported by Haberko et al. that found the concentration of carbonate groups to decrease when the calcination temperature is above 700 °C [40].

The percentage of crystallinity and the crystallite size of the samples were calculated using Scherrer’s equation. Table 3 shows the percentage of crystallinity and the crystallite size of commercial goose bone ash and calcinated goose bone. The calcination process being conducted at a higher temperature causes changes in the crystallite size. Broader diffraction peaks reflect a smaller crystallite size. The crystallite size was bigger for SGBA 900 compared to that for SGBA 300 and CGBA, which might be due to particle coarsening during sintering.

A previous study by Stastny et al. suggested that HA with higher crystallinity tends to have lower solubility and slower degradation rates [41]. Fulmer et al. also investigated the impact of crystallinity on the solubility of the studied ceramics. Among the investigated apatite materials with reduced crystallinity and carbonate substitutions, sintered hydroxyapatite characterized by high crystallinity was the least soluble [42].

### 3.4. FESEM Analysis

**Figure 3:**
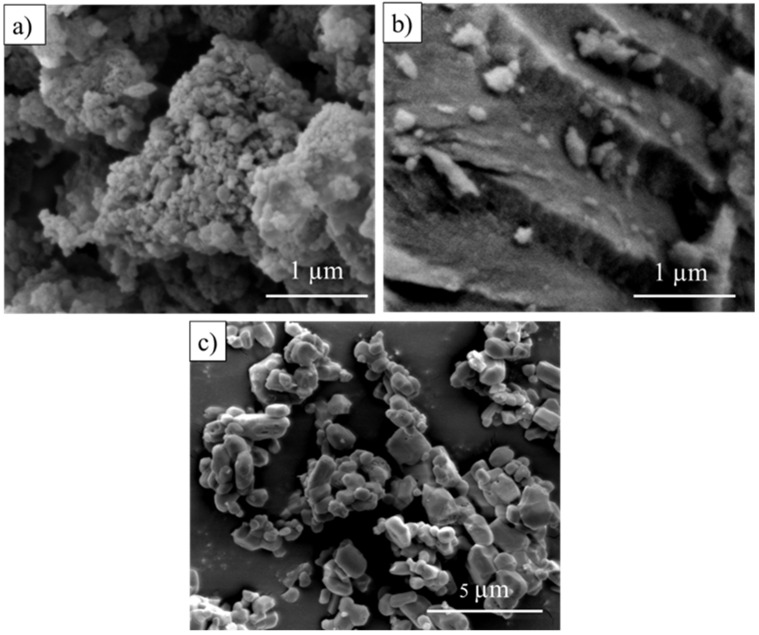
FESEM image of (**a**) CGBA, (**b**) SGBA 300 and (**c**) SGBA 900 at different magnifications.

The investigation of the morphology and size of GBA powders was carried out via FESEM analysis as shown in Figure 3.

Figure 3a–c demonstrate the images of the GBA samples under a resolution/magnification of 80,000 and 50,000, respectively. The result shows the changes in the surface morphology of the commercial bone powder and the studied bone powder at different temperatures. As shown in Figure 3a, the particles of CGBA exhibit the formation of highly agglomerated particles. This bone powder had no well-defined shape with an irregular size. The cotton-shaped nanoparticles of the GBA samples were observed via FESEM imaging. The range of nanoparticles of CGBA was detected to be between 23 nm and 67 nm through FESEM imaging. The broader size distribution of CGBA may have contributed to the random aggregation of nanoparticles, resulting in space formation between nanometer-sized particles [43]. Consequently, an irregular porous structure was created, and this corresponded with the semi-crystalline phase of the electron diffraction pattern (Figure 2). A previous study also reported that there were changes in the surface morphology of the bone powder during the calcination process. A high temperature disintegrated the organic and inorganic phase of the sample powder; thus, multiple pores with a more compact microstructure were able to form [44]. This finding correlates with that of the morphological property of SGBA 300 (Figure 3b) which indicates the dense and thick structure of the sample. These denser nanoparticles (>10 nm) had a smooth texture with a sharp edge appearance.

Meanwhile, nanoparticles of SGBA 900 (Figure 3c) were measured (>100 nm) and structured as crystallite particles. The decomposed bone powder was also observed to be aggregated as coral reefs and appeared as distinct nanoparticles. Hoque et al. and Odusote et al. found out that the temperature of calcination affects the transformation of particle sizes [44,45].

At a high temperature (>650 °C), ab enlargement of particle sizes was discovered, and this may be related to the absorption of heat energy by the particles [45]. Scaffolding materials such as nanohydroxyapatite can have potential in strengthening the mechanical properties of bone substitutes as well as promoting the process of osteogenesis of the osteoblast cell [46].

**Figure 4:**
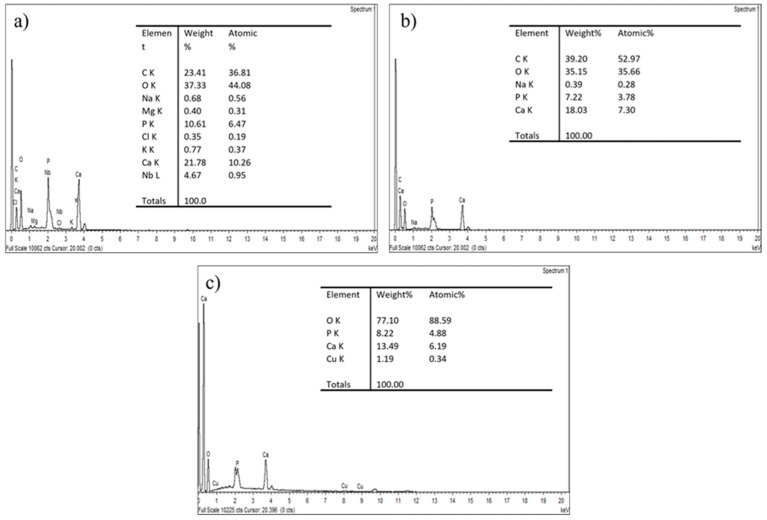
EDX spectrum of (**a**) CGBA, (**b**) SGBA 300 and (**c**) SGBA 900.

Through the EDX spectrum shown in Figure 4, the existence of two main peaks corresponded to the elements Ca and P for every GBA. The Ca/P ratios of CGBA and both SGBA samples were obtained through FESEM/EDX semiquantitative chemical analysis. The calculated ratios for CGBA, SGBA 300 and SGBA 900 were approximately 2.05, 2.50 and 1.64, respectively. These values follow a similar trend and are close to those values from the XRF data. The SGBA 900 that was thermally treated and sintered at 900 °C showed a Ca/P ratio that appeared to be very close to the stoichiometric value, which indicates an optimum thermal temperature. This value is comparable to the ratio of calcified goose beak bone studied by Kim et al. which was reported to be 1.63 [19]. In contrast, CGBA and SGBA 300 had a higher Ca/P ratio compared to the stoichiometric HAp value of 1.67. This might have been a result of the CaO produced during the calcinations [47].

This result is consistent with that of the XRD analysis (Figure 2), which demonstrated the level of the crystalline phase in the GBA samples’ structures. SGBA 900 is considered pure HAp due to its smaller variation in calcium/phosphorus atomic ratios (Ca/P). The range of the Ca/P ratio of 1.650 to 1.667 plays an important role in the mechanical characteristics of the bioceramic materials because different values will affect the treatment behavior [48]. The physicochemical and biological properties of HAp significantly changed when the Ca/P ratio exceeded 1.67. The strength of HAp decreased, and the defects of the HAp crystal structure were inhibited by ionic nuclei such as F^−^, Cl^−^, CO_3_^2−^, Mg^2+^, and Sr^2+^ [49]. However, the greater number of these apatite nuclei enhanced biological activity, compared to the case with pure HAp. Overall, natural HAp has high chemical stability, but has flaws in its mechanical properties which will influence the scaffold’s property.

## 4. Conclusions

In this study, we produced SGBA 300 and SGBA 900 and compared their physicochemical properties to those of commercial goose bone ash (CGBA). The experimental results show that the different temperatures used during calcination revealed the distinguished physicochemical properties of decomposed bone powder. FTIR analysis indicated the existence of an organic compound (an amide group) in CGBA and SGBA 300, but at a higher temperature of calcination (SGBA 900), the absorbance peak of the amide group disappeared. SGBA 900 was found to be highly crystalline via the XRD spectrum, showing a small variation in the Ca/P ratio (1.64) compared to that of other GBA samples. The different variations in the Ca/P ratio obtained through semiquantitative (FESEM/EDX) analysis indicated the different structures of the mineral phase in every GBA powder. In addition, the morphological property of the decomposed bone powder revealed the existence of nanoparticles. CGBA was indicated to have an amorphous structure with greater porosity, whilst SGBA 300 had a compact and dense particle structure. Meanwhile, SGBA 900 presented crystallite particles with a minimally agglomerate structure. In conclusion, the physicochemical properties of SGBA 300 are comparable with those of CGBA, but SGBA 900 shows a significant improvement in mineralogical properties, and the molar ratio of this natural HAp is in the range of that of stoichiometric HAp. Therefore, SGBA 900 is a promising substitute material for bone regeneration. However, the efficacy of the materials in terms of regenerative properties needs to be assessed to warrant their function. Additional in vitro research is needed to evaluate cytotoxicity, osteogenic cell attachment and proliferation to measure immunogenicity, graft resorption, and new bone formation.

## Figures and Tables

**Table 1:** Results of elemental analysis of GBA via XRF (% mass).

	Elemental Content (% Mass)							
Element	Mg	Si	P	S	Cl	K	Ca	Fe
CGBA	0.316	0.407	18.5	0.379	0.913	3.48	75.7	0.381
SGBA 300	-	0.487	16.7	0.363	0.495	1.25	80.6	0.107
SGBA 900	0.641	0.445	20.2	0.0913	0.314	0.328	77.7	0.277

**Table 2:** Phase composition of GBA under different heating processes determined via XRF (% mass).

	Phase Composition (% Mass)							
Element	MgO_2_	SiO_2_	P_2_O_5_	SO_3_	Cl	K_2_O	CaO	Fe_2_O_3_
CGBA	0.413	0.667	31.6	0.652	0.623	2.76	63.0	0.276
SGBA 300	0.385	0.820	29.0	0.677	0.369	1.54	67.0	0.0833
SGBA 900	0.826	0.717	33.9	0.152	0.208	0.256	63.7	0.194

**Table 3:** Percentage of crystallinity and crystallite size of commercial and calcinated GBA.

Samples	Crystallinity	Crystallite Size
CGBA	71.6%	20.0 nm
SGBA 300	62.7%	23.6 nm
SGBA 900	81.4%	38.3 nm

## Data Availability

Not applicable.
